# The relationship between osteoporosis and depression

**DOI:** 10.1038/s41598-022-15248-w

**Published:** 2022-07-01

**Authors:** Seyyed Sadra Kashfi, Gholamreza Abdollahi, Jafar Hassanzadeh, Hamidreza Mokarami, Ali Khani Jeihooni

**Affiliations:** 1 Department of Social Medicine, School of Medicine, Shiraz University of Medical Sciences, Shiraz, Iran; 2 Department of Epidemiology, School of Health, Shiraz University of Medical Sciences, Shiraz, Iran; 3 Department of Ergonomics, School of Health, Shiraz University of Medical Sciences, Shiraz, Iran; 4 Nutrition Research Center, Department of Public Health, School of Health, Shiraz University of Medical Sciences, Shiraz, Iran

**Keywords:** Psychology, Endocrinology, Risk factors

## Abstract

Osteoporosis is the most common metabolic bone disease. The complications of osteoporosis have influence on people's lives and lead to anxiety and depression. The aim of this study was to determine the relationship between osteoporosis and depression among Iranian patients. This cross-sectional analytical survey study conducted among 500 patients referred to a Bone Densitometry Center in Iran. They were assigned into with osteoporosis group (n = 250) and non-affected group (n = 250). The Persian version of the 13-item Beck Depression Inventory (BDI) was used to assess depression. ANOVA, independent t-test, chi-square were used to compare the data. All analyses were done using SPSS version 22 software. A *P* value ≤ 0.05 represented statistical significance. The majority of people with osteoporosis (86.9%), suffered from mild to moderate depression and the majority of normal people (84.6%) were non-depressed. The mean scores (SD) of depressions in the patients with osteoporosis and normal status was 6.94 (2.39) and 2.50 (1.01), respectively. Statistical analyses showed that the mean scores of depressions significantly different between the two groups (*P* < 0.05). The results indicate that depression is associated with osteoporosis. As a result, physicians are expected to pay attention to depression in people with osteoporosis and to treat it.

## Introduction

Osteoporosis is now recognized as a public health problem and is known as the silent disease of the century. It is the most common metabolic bone disease, which appears as bone mass reduction and the resulting complications (fracture), impose high and irreparable financial and physical harm to society and patients^1–5^. The most common and serious complications of osteoporosis are fractures, which is about 1.5 million fractures every year^6,7^. In the United Kingdom and the United States about 7.1 billion pounds and 18 billion dollars a year are spent on these fractures^8^. Also, a pelvic fracture alone in the first year in European countries, costs about 20,000 dollars to treat^9^. Therefore, the importance of this disease is associated with increased fractures in the femur, pelvic and spinal areas^10^.

Factors that increase the chances of osteoporosis in a person include uncontrollable factors, such as familial history of osteoporosis, aging, female gender, menopause, small body and controllable factors such as cigarette smoking, alcohol, low body mass index, sedentary lifestyle, lack of calcium and vitamin D intake, high levels of sodium, protein and caffeine intake, lack of adequate intake of fruits and vegetables, as well as medical and pharmaceutical factors such as long-term corticosteroid use, and diseases such as Rheumatoid Arthritis, thyroid and parathyroid disorders^11–14^.

Chronic illnesses may have several negative consequences that may lead to psychological worries such as depression^15^. The complications of osteoporosis influence on people's lives and lead to anxiety and depression, limitation of activity, acute and chronic pain, difficulty in doing daily routine, dependency to others, and change in social interactions, and ultimately affect the quality of life of suffering people^16^. Depression with some changes in the hormonal system, such as increase in cortisol and decrease in sex hormones, can lead to reduction in bone mineral density^17^.

Depression is one of the most common psychiatric disorders and also the most common mood disorder, which has multi-factorial etiology. It affects 25% of men and 12% of women in their life spans^18,19^. Researches show that every year in the United States about 19 million people experience depression. According to the research conducted in Iran, about 7 million people suffer from a kind of mental disorder, and about 15–25% of the population experience mild to severe depression. This disorder is a common disorder that affects 15% of people at least once in a lifetime. It is also anticipated that the number of depressed people is rising due to pressures from social change, the environment, and some physical illnesses^20^.

Some studies have shown a relationship between depression and osteoporosis. It is reported that people with depression, are at a high risk for osteoporosis^21,22^. Various studies have reported multiple biological mechanisms are associated with both depression and osteoporosis. Depression is associated with a decrease in estrogen and testosterone levels. Additionally, depression is an inflammatory state associated with multiple cytokines such as alpha tumor necrosis factor and IL-6, which results in apoptosis of osteoclasts. In depression, with the disruption of the HPA axis, cortisol plasma levels increase, all of which ultimately leads to reduction in bone density^23^. In another study, it has been shown that in depressed people, levels of 25-hydroxy vitamin D decrease and levels of parathyroid hormone increase^24^, which both can lead to bone loss.

Barbour et al. reported that there is a relationship between increasing inflammatory markers and pelvic fracture^25^. The mechanism of TCA anti-depressant drugs is not clear in bone density reduction, but the complications of these drugs, such as orthostatic hypotension, vertigo and balance disturbance, can lead to falling down and bone fractures^26^. Studies have shown that bone marrow cells have serotonin 5-HT2 receptors. Serotonin plays a key role in bone metabolism. So, the use of SSRI drugs in depressed patients leads to bone density reduction^27^.

Considering the contradictory results of studies on the association between depression and osteoporosis, the present study was conducted to determine the relationship between depression and osteoporosis to improve the health of the community.

## Methods

### Study design and participants

This cross-sectional analytical survey study conducted among patients referred to a Bone Densitometry Center in Iran. To calculate sample size, we used $n = \frac{{\left( {Z_{1 - \alpha /2} \cdot \sqrt {2\overline{P}(1 - \overline{P})} + Z_{1 - \beta } \cdot \sqrt {P_{1} (1 - P_{1} ) + P_{2} (1 - P_{2} )} } \right)^{2} }}{{d^{2} }}$, where $\overline{P}$ = 41.5, $Z_{1 - \alpha /2}$ = 95%, $Z_{1 - \beta }$ = 80%, P1 = 40%, P2 = 43%, d = 0.05. Based onminimum sample size of the obtained 290. In order to improve the power of study, we selected 500 patients (250 patients in each group). In this study, randomized sampling was used; so that among all individuals referring to the bone densitometry center, 250 patients with diagnosis of osteoporosis, were selected as osteoporosis group. Also, to determine the non-osteoporotic group, among the list of referrals to the center, 250 patients who confirmed not having osteoporosis, had been randomly selected as the normal group. Inclusion criteria included persons aged 35 years and older, no history of corticosteroid use for more than 6 months, no history of anticonvulsant use, no history of liver, renal, pulmonary, thyroid, parathyroid and skeletal diseases, insulin-dependent diabetes, autoimmune diseases such as rheumatoid arthritis, no history of osteoporosis treatment, no history of hysterectomy and ovariectomy before menopause, no history of precocious menopause, no history of infertility and no eating disorders. The aims of the study and procedure of the research was explained to the patients, participation was entirely voluntary, written consent was obtained, and the questionnaires were submitted anonymously. The patients filled out the questionnaire in a private and quiet room on the center. The Scientific and Ethics Committee of Shiraz University of Medical Sciences approved the research project (IR.SUMS.MED.REC.1398.308).

### Measures

The survey was made up of the following questionnaires:

#### Demographic information

The following demographic information were measured by a research-made questionnaire: sex, age, BMI, education level, job type, smoking, alcohol consumption, taking calcium, income status, and history of fracture.

#### Depression

The Persian version of the 13-item Beck Depression Inventory (BDI) was used to assess depression. This form is one of the best Standard questionnaires used to evaluation of depression. The BDI is an international standard which was invented by an American psychologist Dr. Beck in 1960^28^. The satisfactory psychometric properties of the Persian version 13-item BDI used have been reported by Rajabi et al.^29^. The Cronbach's alpha and split-half coefficient for the questionnaire were 0.82 and 0.89^30^. In this questionnaire, the score for each question is 0–3 and the score of 21 is the highest score. The score of 0–4 consider as non-depressed, the score of 5–7 as mild depression, score of 8–16 moderate depression, and over 16 consider as severe depression^31^.

### Data analysis

All analyses were done using SPSS version 22 software (SPSS Inc., Chicago, IL, USA). A *P* value ≤ 0.05 represented statistical significance. The Kolmogorov–Smirnov (K–S) test was used for assessment of data distribution and ssumption of normality. The dependent variables were all normally distributed. Thus, To describe quantitative variables, we used mean and standard deviation, and qualitative variables were characterized by frequency and frequency percentage, as well as independent t-test, Chi-square, ANOVA were used to compare the data.

### Ethical approval and consent to participate

The study procedures were carried out following the Declaration of Helsinki. This study was approved by the Ethics Committee of Shiraz University of Medical Sciences. There was an emphasis on maintaining privacy in keeping and delivering the information accurately without mentioning the names of the participants. The participants were given the right to leave the interview at any time if they wished to leave the interview process, and they were promised to have the study results if they want. Informed consent was taken from all the participants. For illiterate people involved, informed consent from a parent and/or legal guardian obtained in the study.

## Results

In this study, 500 people referred to the Bone Densitometry Center were studied. 368 (73.6%) were female and 132 (26.4%) were male. More than half of them had a high school diploma and consisted of domestic women. Tables 1 and 2 shows demographic information of the participants. The distributions of sex, age, BMI, education level, job type, smoking, alcohol consumption, taking calcium, income status, and history of fracture did not significantly differ between the two groups.

**Table 1:** Demographic characteristics of osteoporosis and normal groups.

Variable	Osteoporosis		Normal		P*
	Frequency	Percent	Frequency	Percent	
**Sex**					
Men	188	75.20	180	72	0.56
Women	62	24.80	70	28	
**Education level**					
Illiterate	25	10	20	8	0.32
Under the diploma	134	53.60	142	56.80	
Diploma and beyond the diploma	91	36.40	88	35.20	
**Job type**					
Employee	34	13.60	38	15.20	0.28
Self-employment	64	25.60	68	27.20	
Housewife	152	60.80	144	57.60	
**Smoking**					
Yes	29	11.60	32	12.80	0.61
No	221	88.40	218	87.20	
**Alcohol consumption**					
Yes	2	0.80	1	0.40	0.36
Sometimes	34	13.60	31	12.40	
No	214	85.60	218	87.20	
**Taking calcium**					
Have	95	38	160	64	0.07
Have not	155	62	90	36	
**Income status (Rial)**					
8000/000	48	19.20	45	18	0.37
8000/000–20/000/000	134	53.60	125	50	
20/000/000–50/000/000	26	10.40	32	12.80	
More than 50/000/000	12	4.80	10	4	
No income	30	12	38	15.20	
**History of fracture**					
Have	26	10.40	12	4.80	0.08
Have not	224	89.60	238	95.20	

*Chi-square test.

**Table 2:** Frequency distribution of participants in two groups of osteoporosis and normal in terms of age and body mass index (BMI).

Variable	Group	Range	Mean (M)	Standard deviation (SD)	P*
Age	Osteoporosis	36–68	50.22	7.58	0.12
	Normal	36–69	6.20	48.62	
BMI (kg/m^2^)	Osteoporosis	13.96–18.33	25.08	3.12	0.25
	Normal	17.33–87.77	24.87	3.44	

*BMI* body mass index.

*Independent t-test.

Statistical analyses showed that there was no significant relationship between depression and BMI, sex, education level, job type,smoking, alcohol consumption, taking calcium, income status, and history of fracture variablesin osteoporosis patients (*P* > 0.05). But, pearson correlation coefficient test showed that there was a significant relationship between depression and age in osteoporosis patients (*P* < 0.05) (Table 3).

**Table 3:** Demographic characteristics of osteoporosis patients and their associations with the depression (n = 250).

Variable	Depression	
	Mean (SD)	*P*
Age	6.94 (2.39)	0.02^†^
BMI	6.94 (2.39)	0.64^†^
**Sex**		
Men	7.01 (2.23)	0.56^‡^
Women	6.76 (2.15)	
**Education level**		
Illiterate	6.72 (2.34)	0.27^‡^
Under the diploma	6.97 (2.17)	
Diploma and beyond the diploma	7.13 (2.19)	
**Job type**		
Employee	6.98 (2.17)	0.44^‡^
Self-employment	6.82 (2.41)	
Housewife	6.71 (2.29)	
**Smoking**		
Yes	6.96 (2.06)	0.61^‡^
No	6.74 (2.15)	
**Alcohol consumption**		
Yes	7.21 (2.24)	0.25^‡^
Sometimes	6.82 (2.13)	
No	6.54 (1.95)	
**Taking calcium**		
Yes	6.92 (2.25)	0.78^‡^
No	6.80 (2.17)	
**Income status (Rial)**		
8000/000	7.04 (2.10)	0.83^‡^
8000/000–20/000/000	6.99 (2.23)	
20/000/000–50/000/000	6.90 (2.03)	
More than 50/000/000	6.84 (2.17)	
No income	7.14 (2.39)	
**History of fracture**		
Yes	7.14 (2.11)	0.08^‡^
No	6.54 (2.04)	

*BMI* body mass index, *SD* standard deviation.

^†^Pearson correlation coefficient.

^‡^Independent t test.

^‡^One-way analysis of variance.

The mean scores (SD) of depressions in the patients with osteoporosis and normal status was 6.94 (2.39) and 2.50 (1.01), respectively. It should be noted that according to the standardized scores in BDI, a higher score indicates more severe depression in a person. Statistical analyses showed that the mean scores of depressions significantly different between the two groups (*P* < 0.001). The majority of people with osteoporosis (86.9%), suffered from mild to moderate depression and the majority of normal people (84.6%) were non-depressed.

## Discussion

Regarding the comparison of the frequency distribution of demographic variables in two groups of people with osteoporosis and normal people, the findings showed that the two groups had the same demographic and underlying characteristics (age, level of education, sex, etc.), Which suggests the homogeneity of the studied groups. Study of Saei Gharenaz et al.^18^ is consistent with the results of this study.

The findings of this study indicate that there is a significant difference between bone density status and depression. In other words, people with osteoporosis have higher depression scores than normal people. In recent years, a number of studies have examined the relationship between depression and bone loss, which has controversial results.In the study of Cizza^32^, patients with major depressive disorder had lower bone density compared with the control group and the frequency of osteoporosis was higher. Eskandari et al.^33^ also reported in a study of pre-menopause women that depression is associated with a decrease in bone density. Michelson et al.^16^ also found significant correlation between depression and bone density in a case–control study in 24 women with major depression and 24 without depression. In another study, depressive disorder was associated with reduced bone mineral density in the spine, femoral neck and femur^34^. All of the above studies are consistent with the present study. But some researchers also reported contradictory results. The study of Saei Gharenaz et al.^18^ on Iranin women showed that depression was not significantly associated with osteoporosis. Ljubicic Bistrovic et al.^21^ and Ozsoy et al.^31^ also reported there is not a relationship between osteoporosis and depression which is not consistent with the results of this study. It seems the design method of the study and the study group and important behavioral factors such as lifestyle, culture, history of hormone therapy and the important factor of genetics can be the cause of this contradiction.

The results of this study showed that there is no relationship between depression and sex in people with osteoporosis. In this regard, Ozsoy et al.^31^ reported that there is no relationship between depression and bone loss in men and women in terms of gender. In studies by Patti et al.^35^ and Fallah et al.^36^, there was no statistically significant relationship between depression and sex, which confirms the findings of this study. The results of other studies also confirm this finding^37–39^. Oh et al.^40^ concluded in their study of Korean women aged 80–80 years that depression was associated with a decrease in bone density in men, but in women, depression did not correlate with decreased bone density, which is not consistent with the present study. The causes of this contradiction can be explained by the difference in age group and differences in the culture of the population under study.

The results of studies by Musarezaie et al.^39^, Fallah et al.^36^ and Chevalier et al.^37^ showed that there is no significant relationship between depression and age, which is not consistent with the present study. The heterogeneity of the research samples, the data collection tool, the difference in sample size, and the different entry and exit criteria in studies can be considered for possible reasons for this discrepancy.

The results of this study indicated that there was no correlation between depression and education level in osteoporosis patients. Studies by Musarezaie et al.^39^, Fallah et al.^36^ and Chevalier et al.^37^ suggest that there is a significant relationship between depression and staff educational status, which was not consistent with the present study. The heterogeneity of the research samples, the differences in the data collection tool, the differences in sample size, and the different entry and exit criteria in studies can be considered as the possible reasons for these inconsistencies.

The results of this study showed that there is no significant relationship between depression and job type and income status in osteoporosis patients. In the study of Khajavi et al.^41^, there was a significant negative relationship between employment and economic status with depression, meaning that with increasing employment and improving the economic status of participants in the study, the rate of depression decreased. In addition, there was no significant relationship between depression and history of fracture. Studies have suggested that many drugs prescribed for the treatment of depression, in addition to increasing the likelihood of falling down and thereby increasing the risk of fracture, also affect calcium metabolism. One study reported that depression was 3 times higher in patients with pelvic fracture than in the control group^42^, which is not consistent with the present study.

The results of this study showed that there was no correlation between depression and smoking in sin osteoporosis patients. Smoking is one of the factors affecting bone density^43^. Many studies indicate that there is a high rate of depression and anxiety in smokers^44,45^, which contrasts with the present study.

The limitations of the present study, included not being possible to generalize the study to the whole society as samples were selected from those referring to bone densitometry center; so that it may not present the general population as well. Answering and completing the questionnaire in this project considered as the satisfaction of participating in the study and the participants were assured that their information would stay confidential. Case–control” design used in this study is appropriate to confirm the well-known associations between osteoporosis and depression. However, such design it is not enough for proving that depression increases risk of osteoporosis. In addition, "clinical” sample as a control group can lead to bias. The participants of "main" group with the diseases forced them to seek medical help at the osteoporosis center, more likely could have symptoms such as fatigue, pain, weakness that may artificially inflate BDI-13 scores due to symptoms of the illness, rather than of depression. Despite, the study could be consider as a pilot for future wide population-based research in original population of Iran as well as the clinical research in osteoporosis.

## Conclusion

According to the results of this study, which depression is associated with osteoporosis, depression should be considered as a risk factor for osteoporosis, such as smoking or low calcium intake. Also, the results of this study can be used as a guide for implementation of educational program in people with osteoporosis, in order to help their patients and their families to promote mental health, especially control and reduce depression.

## Data Availability

The datasets used and/or analysed during the current study are available from the corresponding author upon reasonable request.

## Abbreviation

BMI: Body mass index
